# Secretory leukocyte protease inhibitor suppresses HPV E6-expressing HNSCC progression by mediating NF-κB and Akt pathways

**DOI:** 10.1186/s12935-019-0942-7

**Published:** 2019-08-23

**Authors:** Yu Jin, Yuexiu Li, Xin Wang, Ya Yang

**Affiliations:** 1 Department of General Dentistry, Ninth People’s Hospital, Shanghai Jiao Tong University School of Medicine, 639 Zhizaoju Road, Shanghai, 200011 P. R. China; 2 Shanghai Key Laboratory of Stomatology and Shanghai Research Institute of Stomatology, National Clinical Research Center of Stomatology, Shanghai, 200000 P. R. China; 3 Department of Stomatology, Tai’an Central Hospital, Tai’an, Shandong 271000 P. R. China

**Keywords:** Head and neck squamous cell carcinoma, Human papillomavirus, Secretory leukocyte protease inhibitor, NF-κB, Akt pathway

## Abstract

**Background:**

Head and neck squamous cell carcinoma (HNSCC) is the sixth most common cancer worldwide and human papillomavirus (HPV) has been increasingly recognized as a pathogenic factor for the initiation and development of HNSCC. E6 oncogene, an essential component of the HPV 16 virus, acts as a leading cause of the malignant transformation of cancer cells. Therefore, investigating the biological effect and potential mechanisms of E6 oncogene on HNSCC cells and exploring potential therapeutic methods is of great value.

**Methods:**

MTT assay, cell cycle analysis, and apoptosis assay were implemented to detect the biological effect of E6 oncogene on the growth of HNSCC cells. Wound healing assay and transwell assay were used to evaluate the role of E6 in the migration and invasion of HNSCC cells. Western blot and immunofluorescence assay were adopted to explore the regulatory mechanisms underlying E6-induced HNSCC progression. Then, exogenous secretory leukocyte protease inhibitor (SLPI) was added into the cell culture to investigate whether it could maintain its tumor suppressor effect on E6-expressing HNSCC cells.

**Results:**

HPV E6 oncogene could promote the proliferation, cell cycle period, apoptosis resistance, migration and invasion of HNSCC cells by activating NF-κB and Akt pathways. Immunohistochemical analysis conducted on HNSCC tissues illustrated that SLPI was further downregulated in HPV positive HNSCC compared to HNSCC without HPV infection. Exogenous SLPI significantly inhibited HPV E6-mediated malignant phenotypes in HNSCC cells by inhibiting the activation of NF-κB and Akt and signaling pathways.

**Conclusions:**

This study demonstrated that E6 oncogene led to the malignant transformation of HNSCC cells by regulating multiple pathways. SLPI could reverse the effect of E6 oncogene on HNSCC, implying that the functional inhibition of E6 by SLPI may be exploited as an attractive therapeutic strategy.

## Background

Head and neck squamous cell carcinoma (HNSCC) is the sixth most common cancer with approximately 600,000 new cases worldwide [1]. It has been widely acknowledged that tobacco and alcohol consumption are the two most important risk factors for HNSCC, while recently, human papillomavirus (HPV) has been increasingly recognized as a pathogenic factor for the initiation and development of HNSCC. It has been reported that HPV-induced HNSCC cases have increased considerably in recent years [2]. Therefore, investigating the role of HPV in the development and progression of HNSCC and finding essential regulatory mechanisms are of great value.

Over 100 different types of HPV, a group of small nonenveloped DNA viruses, have been identified, and more than 15 of these viruses are considered to have carcinogenic potential [3]. HPV 16 infection has been illustrated to be more prevalent than any other high-risk HPV types in most regions of the world [4, 5]. Additionally, previous studies have reported that HPV 16 is the most common subtype and accounts for nearly 90% of HPV-positive HNSCC. Furthermore, viral E6/E7 oncogenes have been demonstrated to play a central role both in the initiation of HPV-induced carcinogenesis and the malignant growth of HPV-positive cancer cells. Interestingly, the E6 protein could induce ubiquitin-mediated p53 degradation, leading to a significant loss of p53 activity [6]. p53, a tumor suppressor, is able to arrest cells in G1 phase and induce apoptosis activities. Thus, E6-expressing cells are resistant to apoptosis and G1/G2 arrest abilities, resulting in the transformation and deterioration of cancer cells. Therefore, the functional inhibition of E6 may be exploited as an attractive therapeutic strategy. The present study aimed to investigate the effect of HPV 16 E6 protein on the biological characteristics of HNSCC and to identify a feasible method to treat HPV 16 E6 positive HNSCC.

Secretory leukocyte protease inhibitor (SLPI), a nonglycosylated, single-chain protein with a molecular weight of 11.7 kDa, was originally identified as a serine proteinase inhibitor with activities against a variety of proteases [7], and played an important role in the development and metastasis of multiple cancers, such as liver cancer [8], breast cancer [9], ovarian cancer [10] and gastric cancer [11]. In our previous study, we identified that SLPI abundance in HNSCC tissues is much lower than that in normal tissues and that SLPI could act as a tumor suppressor by inhibiting proliferation and promoting apoptosis of HNSCC cells [12, 13], suggesting a tumor suppressive role of SLPI in HNSCC. Due to the critical role of E6 oncogene in maintaining the malignant phenotype of HPV-positive cancer cells, we wondered whether SLPI could still maintain its inhibitory role in HPV E6 positive HNSCC cells. Moreover, a study involving 307 HNSCC cases exhibited an inverse correlation between HPV infection and SLPI expression [14], and HNSCC patients with HPV infection always showed no or low SLPI expression [15, 16]. The above data revealed that SLPI may play significant roles in the progression of HPV-associated HNSCC.

Hence, the present study will investigate the functional role of E6 oncogene in HNSCC progression and identify whether SLPI could abolish the aggressive phenotypes induced by E6 oncogene.

## Methods

### Tissue samples

A total of 24 HPV positive HNSCC tissues and 28 HPV negative HNSCC tissues were obtained from the patients who were diagnosed with HNSCC and underwent surgery at the Department of Oral Maxillofacial‐Head and Neck Oncology, Ninth People’s Hospital, Shanghai Jiao Tong University School of Medicine (Shanghai, China). All of the tissue samples were frozen in liquid nitrogen for further experiments. This study was approved by the Review Board of the Medical Ethics Committee of the Ninth People’s Hospital, Shanghai Jiao Tong University School of Medicine. Informed consent and approval were received from all patients.

### Cell culture

The human HN4 and HN30 cells were generously provided by the University of Maryland Dental School, USA. 293T cells were purchased from the American Type Culture Collection (ATCC, USA). All the cells were cultured in DMEM (GIBCO-BRL, USA) supplemented with 10% fetal bovine serum (GIBCO-BRL, USA) and 1% penicillin and streptomycin at 37 °C, 5% CO_2_ in a humidified atmosphere.

### Plasmid construction and stable transfection

The cDNA of HPV 16 E6 was amplified by high-fidelity PCR (PrimeSTAR; Takara, Japan) with one set of primers (forward primer: 5′-TGTAAAACGACGGCCAGT-3′ and reverse primer: 5′-CAGGAAACAGCTATGACC-3′) and subcloned into the EcoR I and BamHI sites of the PGMLV-6395 vector (Genomeditech, Shanghai). To construct HNSCC cells with a stable overexpression of HPV E6, the lentivirus package method was adopted, and detailed procedures were as follows. The 293T cells were transfected with 10 µg pLenti-E6 or pLenti-NC, 5 µg pCMV-VSV-G and 5 µg pCMV-Delta8.9 using Lipofectamine 2000 reagent (11668019, Invitrogen, USA). After approximately 24 h, the culture medium was replaced with 10 mL fresh medium. The supernatants were collected at 72 h after transfection and then filtered by a 0.45 µm cellulose acetate filter (Merck Millipore, USA). To improve the infection efficiency, 10 µg/mL of polybrene (40804ES76, YEASEN, China) was added into the supernatants. Approximately 6–8 h after the lentivirus package, the medium was exchanged with DMEM medium containing 10% FBS. The E6-expressing cells were selected with 10 µg/mL puromycin (60210ES25, YEASEN, China) for 4 weeks before experiments.

### Administration of recombinant human SLPI (rhSLPI) and intake of exogenous SLPI

Recombinant human SLPI (rhSLPI) was purchased from Sino Biological (Beijing) and sterile ddH_2_O was used to dissolve the reagent and stored at − 40 °C until use. Based on our previous studies [12, 13], we selected a concentration of 40 μg/mL to investigate the effect of SLPI on the biological characteristics of E6 positive or negative HNSCC cells. To rule out any hybrid effects, the same volume of ddH_2_O was added into the culture medium as a negative control. To determine whether exogenous SLPI could get internalized into the cells, the TCS SP2 laser-scanning confocal microscope (Leica Microsystems, Germany) was used to observe the localization of SLPI in HN4 cells by immunofluorescent assay. SLPI protein (40 μg/mL) or equal volume ddH2O was added to the cells and cultured for 1 h. Then, the cell nucleus was stained with DAPI (blue), the cytoskeleton was stained with phalloidine (red), and SLPI was stained with FITC secondary antibody (green). Images were captured using Zen-Software (Carl Zeiss AG, Oberckochen, Germany).

### Treatment of NF-κB and Akt pathway specific inhibitors

To further investigate whether the effects of E6 or SLPI on HNSCC are really dependent on NF-κB and Akt signaling, we utilized specific inhibitors of the NF-κB (S1808, PDTC, Beyotime) and Akt pathways (S1078, MK-2206, Selleck). Specifically, cells treated with or without 100 μmol PDTC or 5 μmol MK-2206 were transfected with HPV E6 or NC, and protein was extracted after 72 h. Exogenous SLPI and specific inhibitors were respectively added into cells for 24 h after which the extracted protein was subjected to western blot experiments.

### RNA extraction and quantitative real-time PCR

Total RNA from the cells was extracted using Trizol reagent (9109, Takara, Japan) and complementary cDNA was synthesized by using a PrimeScript™ RT reagent kit (RR047A, Takara, Japan). Real-time PCR was performed on an ABI StepOne real-time PCR system (Life Technologies, USA) by using a SYBR Premix Ex Taq Reagent Kit (RR820A, Takara, Japan). The reaction conditions were as follows: 95 °C for 5 min, 40 cycles of 5 s at 95 °C, 30 s at 60 °C. All the primers were designed and synthetized by Sangon Biotech (Shanghai) and the detailed sequences for the primers are presented in Table 1. GAPDH was used as the internal reference. The relative expression level was calculated by adopting the 2^−ΔΔCt^ method and all experiments were repeated in triplicate.

**Table 1:** The primers used for real-time PCR analysis

Genes	Primer sequences
Human SLPI	Forward 5′-GAGATGTTGTCCTGACACTTGTG-3′
	Reverse 5′-AGGCTTCCTCCTTGTTGGGT-3′
HPV 16 E6	Forward 5′-GAGAACTGCAATGTTTCAGGACC-3′
	Reverse 5′-TGTATAGTTTGCAGCTCTGTGC-3′
Human IL-6	Forward 5′-ACTCACCTCTTCAGAACGAATTG-3′
	Reverse 5′-CCATCTTTGGAAGGTTCAGGTTG-3′
Human IL-8	Forward 5′-TTTTGCCAAGGAGTGCTAAAGA-3′
	Reverse 5′-AACCCTCTGCACCCAGTTTTC-3′
Human IL-1β	Forward 5′-ATGATGGCTTATTACAGTGGCAA-3′
	Reverse 5′-GTCGGAGATTCGTAGCTGGA-3′
Human TNF-α	Forward 5′-CCTCTCTCTAATCAGCCCTCTG-3′
	Reverse 5′-GAGGACCTGGGAGTAGATGAG-3′
Human c-myc	Forward 5′-GGCTCCTGGCAAAAGGTCA-3′
	Reverse 5′-CTGCGTAGTTGTGCTGATCA-3′
Human GAPDH	Forward 5′-ACAACTTTGGTATCGTGGAAGG-3′
	Reverse 5′-GCCATCACGCCACAGTTTC-3′

### Cell proliferation assay

To evaluate the influence of HPV E6 oncogene on the proliferation of HNSCC cells, HN4 and HN30 cells stably transfected with or without HPV E6 were seeded in 96-well plates at a density of 1 × 10^3^ cells/well in triplicate and analyzed for 6 consecutive days. To analyze the impact of SLPI on the growth of HNSCC cells, E6 positive or E6 negative HNSCC cells were plated in 96-well plates (3 × 10^3^ per well). Approximately 24 h later, the cells were incubated with 40 μg/mL exogenous SLPI or the same volume of ddH_2_O and the cell viabilities were analyzed at 24 h, 48 h, and 72 h, respectively. For measurement, the cells were incubated with 100 μL of 0.5 mg/mL MTT (M2003, Sigma-Aldrich, USA) in DMEM medium at 37  °C for 4 h. The formazan that formed was then solubilized by adding 100 μL dimethyl sulfoxide (DMSO). Absorbance was read at the wavelength of 490 nm on a microplate reader to assess relative cell viability. All the experiments were repeated for three times.

### Cell cycle and apoptosis analysis

For cell cycle analysis, tumor cells with or without HPV E6 expression and cells that were administered 40 μg/mL SLPI were harvested, washed by the cold PBS for three times, and fixed with 70% ethanol overnight at 4 °C. The next day, the fixed cells were washed three times using the cold PBS and were then stained with propidium iodide staining buffer (550,825, 50 μg/mL; BD Pharmingen, USA) for around 15 min at room temperature in the dark. Afterwards, the cell cycle distribution of these cells was analyzed using a flow cytometer (BD Biosciences, USA).

The apoptosis assay was performed using an Annexin V, 633 Apoptosis Detection Kit (AD11, DOJINDO, Japan). Briefly, the cells for apoptosis analysis were harvested, washed with cold PBS for three times and made into single-cell suspension in the binding buffer. Then, 5 μL PI staining buffer and 5 μL Annexin V staining buffer were added into the suspension, respectively. After being stained in the dark for about 20 min, cell samples were analyzed with a flow cytometer (BD Biosciences, USA).

### Wound healing assay

Tumor cells with or without HPV E6 expression and cells that were administrated with 40 μg/mL SLPI were plated in the 6-well plates and cultured to achieve 90–100% confluency. Afterwards, cells were scraped with a P200 tip and washed with PBS to remove the floating cells. Cells were incubated with serum-free medium to eliminate the proliferation effect. Pictures were captured at 0 h and 12 h for 5 different fields under an inverted microscope.

### Transwell migration and invasion assay

To investigate the role of HPV E6 oncogene and SLPI in the migratory and invasive abilities of HNSCC cells, transwell chambers (MCEP24H48, Merck Millipore, USA) were coated without or with Matrigel (CB-40234, Corning, USA) on the upper chamber, respectively. Cells were seeded in the upper chamber with a total of 4 × 10^4^ tumor cells in 350 μL serum-free DMEM. The bottom chambers were filled with 700 μL DMEM and 20% FBS was added as a chemoattractant. After incubation for 48 h, cells that had migrated or invaded to the lower surface of the membrane were fixed with 4% paraformaldehyde, stained with 0.5% crystal violet and counted in five non-overlapping fields under a light microscope.

### Immunofluorescence assay

Cells grown on the cover slips were washed with PBS for three times, and then fixed with 4% paraformaldehyde for 30 min. Subsequently, the cells were permeabilized with 0.1% Triton X-100 for 10 min. Afterwards, cells were incubated with 3% H_2_O_2_ for 10 min and blocked with 10% goat serum plus 3% BSA for 30 min. The fixed cells were probed overnight at 4 °C with primary antibodies against HPV 16 E6 (ab70, Abcam, USA; 1:50), p65 (6956, CST, USA; 1:800) and followed by incubation with secondary antibody: Alexa Fluor 594-conjugated goat anti-mouse IgG (AS054, Invitrogen, USA; 1:200) for 1 h at room temperature. The cover slips were treated with DAPI staining dilution (40728ES03, YEASEN, China; 1:500) to detect nuclei. Fluorescence images were finally observed and imaged using a fluorescence microscope (ZEISS, Germany) and TCS SP2 laser-scanning confocal microscope (Leica Microsystems, Germany).

### Immunohistochemical analysis

Paraffin-embedded, 5-μm thick HNSCC sections were deparaffinized, rehydrated, and submerged into citric buffer for microwave-based antigen retrieval and immersed in 0.3% hydrogen peroxide to block endogenous peroxidase activity. Then, they were blocked with 3% bovine serum, after which they were incubated with primary antibodies against SLPI (ab17157, Abcam, USA; 1:50), HPV E6 (ab70, Abcam, USA; 1:50), p65 (6956, CST, USA; 1:800), and p-Akt (Ser473) (4060, CST, USA; 1:100) at 4 °C overnight. The secondary antibody reaction was carried out for 60 min at room temperature. The sections were then stained with hematoxylin, dehydrated, cleared and mounted. Tissues exhibiting brown staining in the cytoplasm, nucleus or membrane were considered positive. Five areas of each section were randomLy (randomly) selected under the same conditions for further analysis. The integrated optical density (IOD) of protein expression was quantitatively calculated using Image-Pro Plus 6.0.

### Western blot analysis

Proteins were extracted from the cells at the indicated times using SDS lysis buffer (P0013G, Beyotime, China). Nuclear and Cytoplasmic Extraction Reagents (P0028, Beyotime, China) were used to separate the cytoplasmic and nuclear extracts from cultured cells. The protein samples (30 μg) were subjected to 4–20% polyacrylamide gels (JC-PE022/JC-PE022R, Genshare biological, China) to be electrophoresed and transferred to 0.22 μm PVDF membranes (ISEQ00010, Merck Millipore, USA) at 90 V for 1.5 h. Membranes were blocked with 5% skim milk for about 1 h at room temperature and then incubated with primary antibodies against HPV 16 E6 (sc-460, Santa Cruz, USA; 1:200), IκBα (4814, CST, USA; 1:1000), p-IκBα (Ser 32/36) (9246, CST, USA; 1:1000), p65 (10745-1-AP, Proteintech, USA; 1:1000), p-p65 (Ser536) (3031, CST, USA; 1:1000), p-p65 (Ser468) (3039, CST, USA; 1:1000), H3 histone (4499, CST, USA; 1:1000), Akt (10176-2-AP, Proteintech, USA; 1:1000), p-Akt (Ser473) (4060, CST, USA; 1:1000), p-Akt (Thr308) (13038, CST, USA; 1:1000), GAPDH (Proteintech, USA; 1:10,000), and β-actin (3700, CST, USA; 1:1000) at 4 °C overnight. Thereafter, the membranes were probed with IR Dye-labeled secondary antibodies (800CW, LI-COR Bioscience, USA; 1:10,000) and the signals were observed using an Odyssey Infrared Imaging System (LI-COR Bioscience, USA).

### NF-κB luciferase reporter gene assay

NF-κB luciferase reporter assay was adopted to evaluate relative NF-κB activity. Briefly, the cells were plated in the 24-well plate and cultured to attain a confluency of 70–80%. After adherence, the cells were cotransfected with 600 ng of NF-κB luciferase reporter gene constructs (Beyotime, China) and 200 ng of *Renilla* luciferase (Beyotime, China), which was used to normalize data for transfection efficiency. After 24 h of transfection, the cells were treated with exogenous SLPI (40 μg/mL) or the same volume of ddH_2_O. The cells were then cultivated for 12 h and cell lysates were analyzed using a dual luciferase reporter assay kit (RG027,Beyotime, China) on Modulus™ (Turner Biosystems, Sunnyvale, CA, USA).

### Statistical analysis

Statistical analysis was performed with SPSS 21.0 software in this study. All numerical data was expressed as mean ± SD from triplicate experiments and comparisons between two or more groups were performed by Student’s two-tailed *t* test or one-way ANOVA. *P* values less than 0.05 were considered statistically significant.

## Results

### Establishment of HPV E6-expressing HNSCC cells

**Fig. 1:**
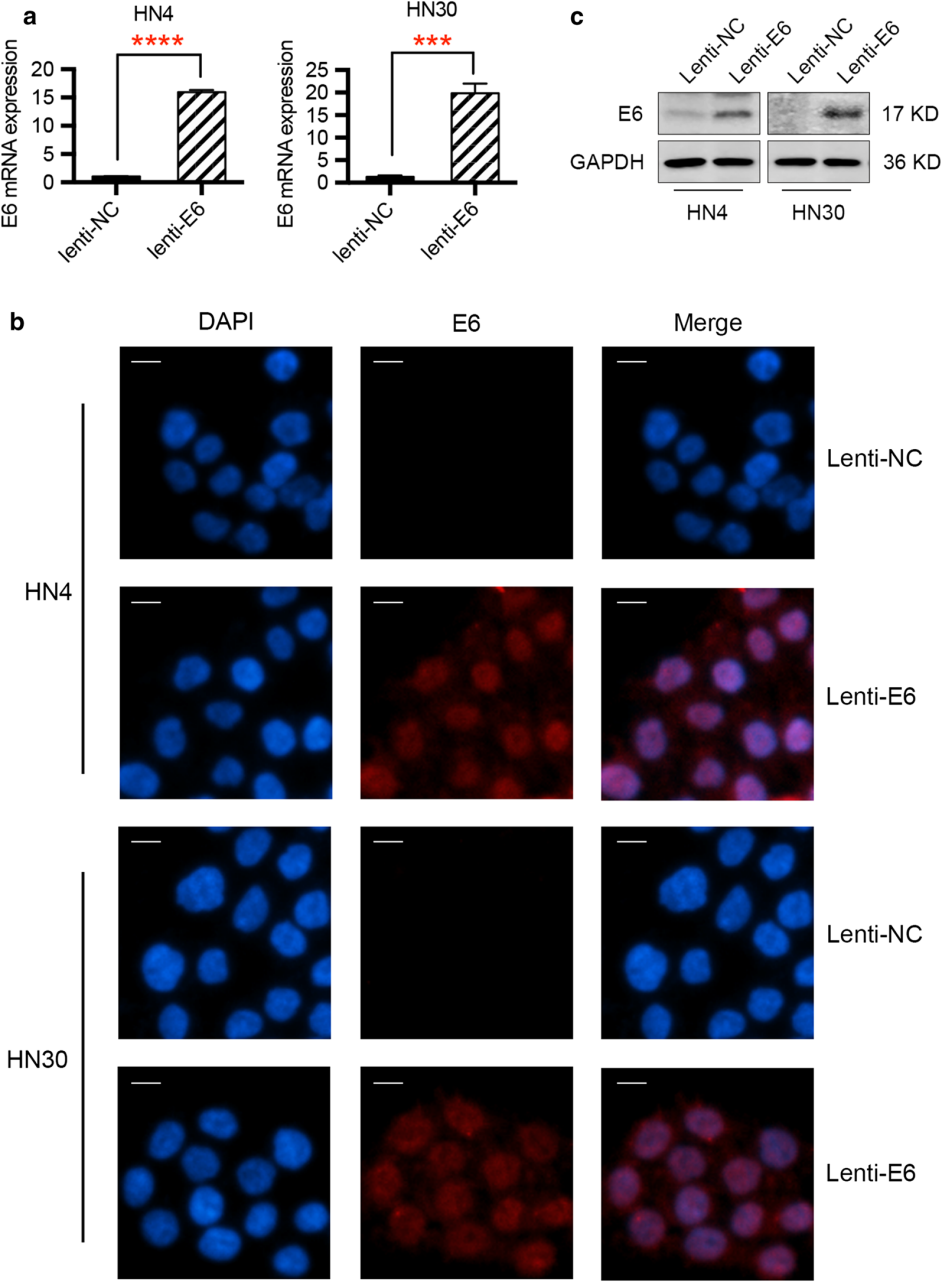
Overexpression of E6 oncogene in HNSCC cells with a stable lentivirus transfection. **a** mRNA level of E6 oncogene was elevated in HNSCC cells with lentivirus transfection, as demonstrated by qPCR technique. **b** Immunofluorescence assay illustrated the elevated protein level of E6 oncogene in HNSCC cells after lentivirus transfection. **c** Western blot results demonstrated the overexpression of HPV E6 oncogene in HN4 and HN30 cells. ***P < 0.001. ****P < 0.0001 (scale bar: 20 μm)

To analyze the functional role of E6 oncogene in HNSCC progression, the establishment of HPV E6-expressing HNSCC cells was needed. Firstly, HN4 and HN30 cells were infected with a lentiviral vector carrying HPV E6 gene. Then, the tumor cells stably expressing HPV E6 were selected with puromycin (10 μg/mL). After the construction of E6 stably expressing HNSCC cells, we determined the overexpression of E6 at mRNA and protein levels. As suggested by Fig. 1a, HN4 cells with a stable transfection of E6 presented approximately 15-fold E6 mRNA overexpression when compared to E6 negative cells, while the lenti-E6 infection resulted in about 20-fold overexpression of E6 oncogene in HN30 cells. Immunofluorescence assay demonstrated that E6 protein was expressed in HNSCC cells after lentivirus transfection (Fig. 1b). Western blot results also illustrated that E6 oncogene was overexpressed in HN4 and HN30 cells (Fig. 1c). The above data revealed that we successfully established HPV E6-expressing HNSCC cells.

### HPV E6 oncogene influences the biological characteristics of HNSCC cells in vitro

**Fig. 2:**
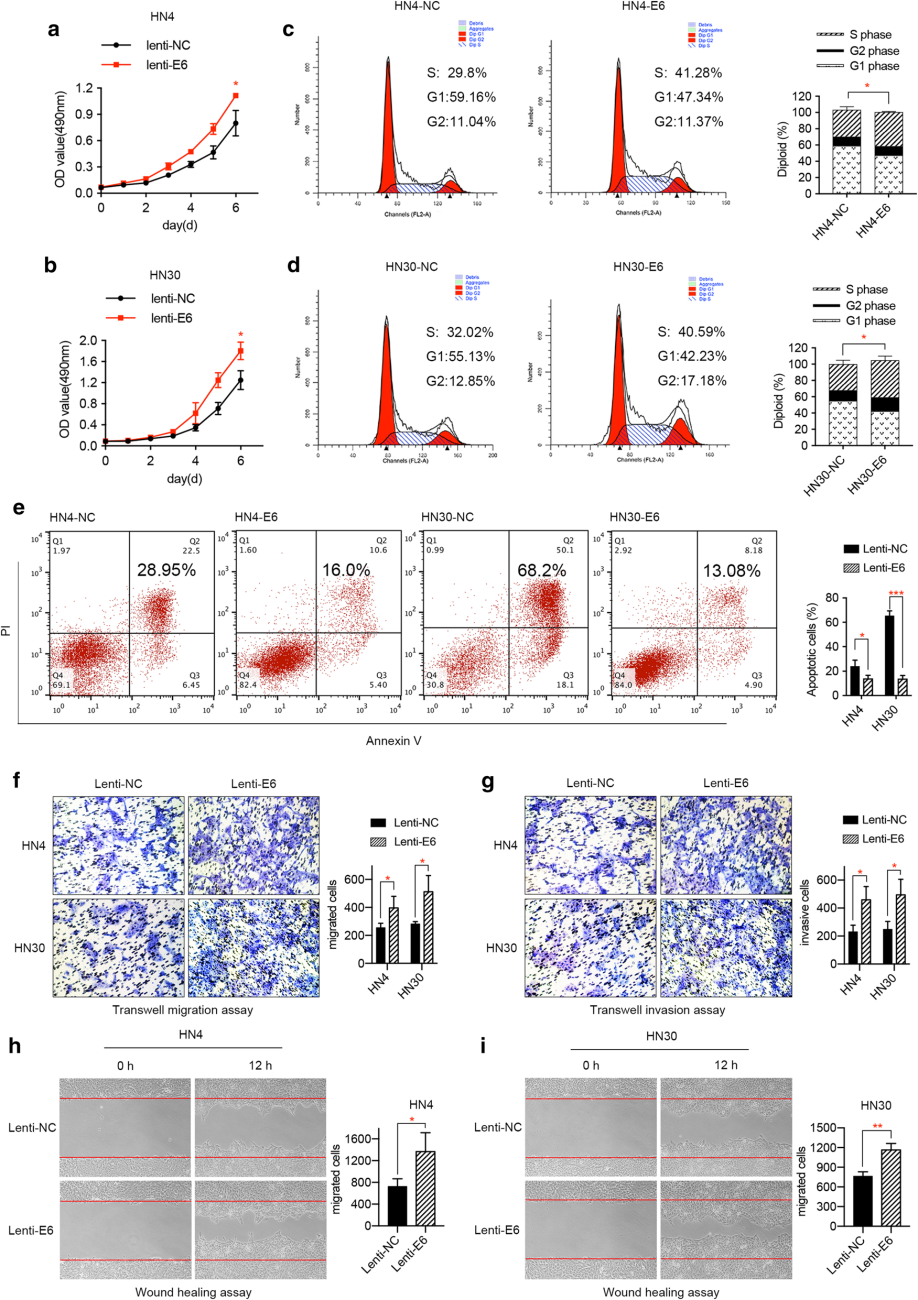
E6 oncogene promotes the proliferation, migration, and invasion of HNSCC cells. **a**, **b** MTT experiment illustrated that E6 oncogene facilitated the proliferation of HNSCC cells. **c**, **d** Cell cycle assay demonstrated that E6 promoted cell growth by increasing the number of cells in S phase and decreasing cells in G1 phase. **e** Apoptosis assay results showed that E6 oncogene significantly resisted the apoptosis activities induced by DMSO in HNSCC cells. **f** Transwell migration assay revealed E6 oncogene facilitated the migration of HNSCC cells. **g** Transwell invasion assay illustrated E6 oncogene significantly elevated the invasive abilities of HNSCC cells. **h**, **i** E6 oncogene enhanced the migrating capacities of HNSCC cells, suggested by wound healing assay. The fields of migrated and invasive cells on the membrane were captured (magnification × 100). Statistical analysis was performed using the t-tests. The data represented the mean values of three independent experiments. *P < 0.05. **P < 0.01. ***P < 0.001

Due to previous findings that E6 oncogene may account for the malignant transformation of cancers, we aimed to investigate whether it could affect the proliferation of HNSCC cells. Firstly, MTT assay was performed to evaluate the effect of E6 oncogene on the proliferation of HNSCC cells. As a result, the growth rates of HN4 and HN30 cells with stable E6 expression were significantly higher when compared to control cells (Fig. 2a, b). Moreover, flow cytometry analysis revealed that E6 oncogene influenced cell cycle distribution to a great extent, mainly manifested by the increase of cancer cells in the S phase and the decrease of cells in the G2 phase (Fig. 2c, d). In addition, cell apoptosis assay was implemented to demonstrate the role of HPV E6 on the apoptosis activity of HNSCC cells. As shown in Fig. 2e, the number of apoptotic cells induced by DMSO was markedly decreased after the HNSCC cells were transfected with E6 oncogene. Therefore, we concluded that HPV E6 promoted the proliferation, cell cycle period and apoptosis resistance of HNSCC cells, thus accelerating the growth of HNSCC.

To investigate whether HPV E6 can play a role in the migration and invasion of HNSCC cells, transwell assay and wound healing experiment were adopted to assess the metastatic ability of HNSCC cells. As presented by the pictures captured by the microscope, overexpression of HPV E6 oncogene remarkably promoted the migratory and invasive capacities of both HN4 and HN30 cells (Fig. 2f, g). In accordance with the above data, enhanced cell motility was observed in E6-expressing HNSCC cells using wound healing assay (Fig. 2h, i). These results revealed the tumor promoting role of HPV E6 in the migration and invasion of HNSCC cells.

### E6 oncogene activates NF-κB and Akt signaling pathways in HNSCC cells

**Fig. 3:**
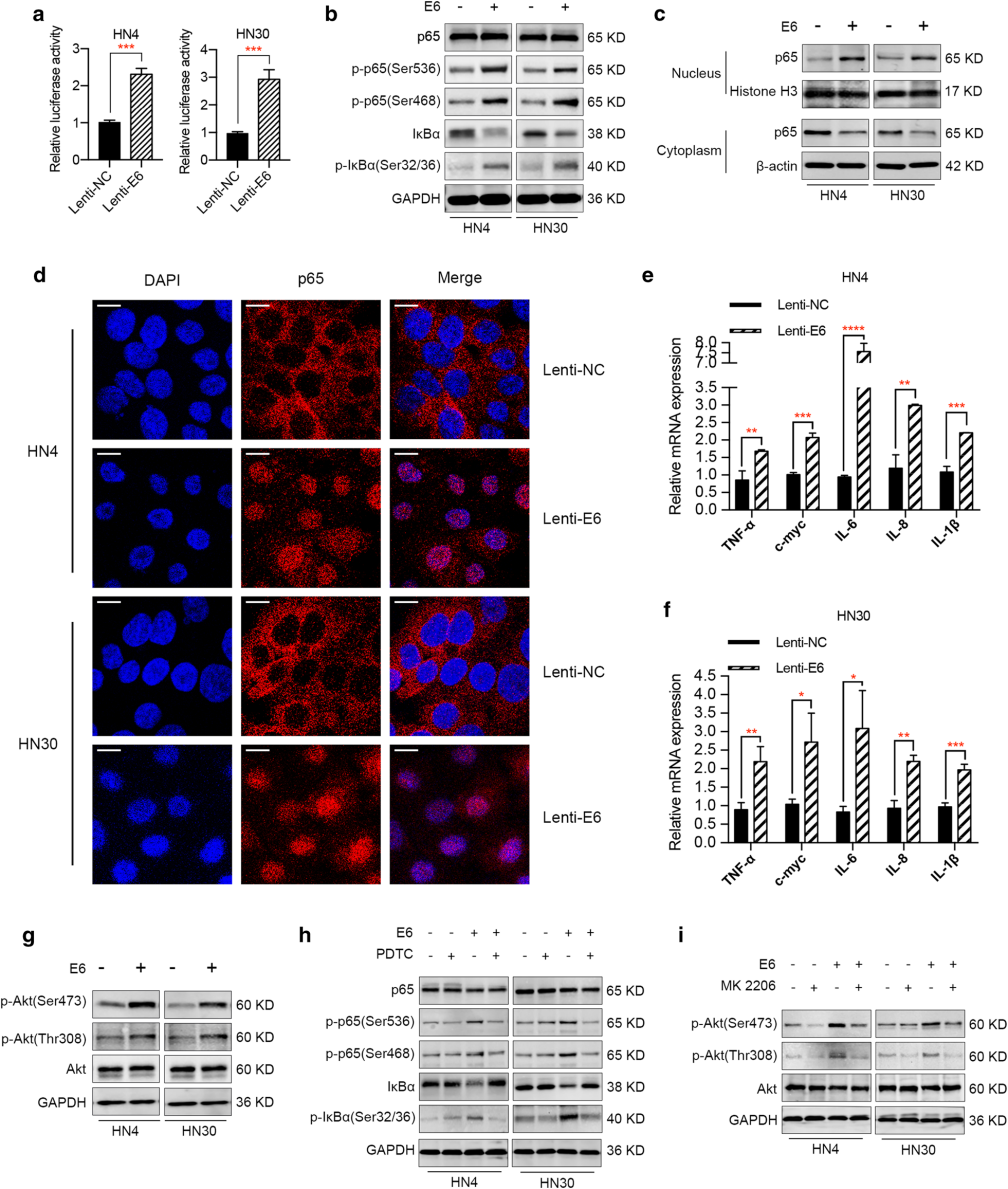
E6 oncogene activates NF-κB and Akt pathways in HNSCC. **a** NF-κB luciferase reporter assay demonstrated an increase of NF-κB activities in E6-expressing HNSCC cells, suggesting the activation of NF-κB pathway by E6 oncogene. **b** Western blot results illustrated that E6 oncogene activated NF-κB pathway and regulated signaling-related proteins expression. **c** Western blot analysis revealed that p65 was localized mostly in the cytoplasm in E6 negative HNSCC cells, while p65 was translocated from the cytoplasm to the nucleus in E6-expressing cells. **d** Confocal microscopy analysis confirmed the nuclear accumulation of p65 in E6-expressing HNSCC cells. **e**, **f** mRNA levels of several key pro-inflammatory NF-κB-dependent cytokines and genes TNF-α, IL-1β, IL-6, IL-8 and c-myc were elevated in E6 positive HNSCC cells. **g** E6 oncogene activated Akt signaling pathway in HNSCC, demonstrated by promoting the phosphorylation of Akt protein. **h**, **i** Specific inhibitors of NF-κB (PDTC, Beyotime) and Akt pathways (MK-2206, Selleck) were utilized to demonstrate the effect of E6 oncogene on HNSCC was really dependent on these two pathways. *P < 0.05. **P < 0.01. ***P < 0.001. ****P < 0.0001 (scale bar: 10 μm)

A variety of studies have demonstrated that HPV infection can activate NF-κB signaling pathway while others maintain that HPV infection inhibits the activation of NF-κB pathway. To investigate the effect of E6 on the activation of NF-κB signaling pathway in HNSCC, a NF-κB luciferase reporter assay was conducted to evaluate the relative NF-κB activities in E6-expressing cells. As shown in Fig. 3a, ectopic expression of E6 oncogene resulted in approximately two to threefold upregulation of NF-κB activities in HN4 and HN30 cells, suggesting the activation of NF-κB signaling pathway by E6 oncogene. Moreover, we detected the expression of NF-κB signaling pathway-related proteins by western blotting. Based on previous studies [17, 18], we selected p65, p-p65, IκBα and p-IκBα for further analysis. As shown in Fig. 3b, E6 oncoprotein could promote the phosphorylation of p65 and IκBα and trigger the degradation of IκBα in HNSCC cells. To further demonstrate the activation of NF-κB pathway by E6 oncogene, we separated nuclear and cytoplasmic protein from E6 positive and E6 negative HNSCC cells. Western blot analyses revealed that p65 was localized mostly in the cytoplasm in E6 negative HNSCC cells, while p65 was translocated from the cytoplasm to the nucleus in E6-expressing cells (Fig. 3c). Accordingly, confocal microscopy analysis visually confirmed the nuclear accumulation of p65 in E6-expressing HNSCC cells (Fig. 3d). In addition, we also detected the mRNA levels of several key pro-inflammatory NF-κB-dependent cytokines and the genes TNF-α, IL-1β, IL-6, IL-8 and c-myc [19, 20]. As suggested by Fig. 3e and f, the mRNA levels of the above cytokines and genes underwent an upregulation in E6 positive HNSCC cells, confirming that the activation of NF-κB signaling pathway promoted the transcription of downstream genes. Overall, the above results revealed that E6 oncogene activated NF-κB signaling pathway in HNSCC. Apart from NF-κB signaling pathway, HPV infection has also been reported to affect the activity of Akt pathway, which is closely associated with HNSCC progression [21, 22]. Therefore, we tried to investigate the potential effect of E6 oncogene on Akt pathway. As a result, western blot analysis showed that E6 oncoprotein promoted the phosphorylation of Akt while no significant changes in the total Akt protein were observed in HNSCC cells overexpressing E6 (Fig. 3g). To further investigate whether the effects of E6 on HNSCC are really dependent on NF-κB and Akt signaling, we utilized PDTC and MK-2206, which are specific inhibitors of NF-κB and Akt signaling, respectively. Specifically, cells treated with or without 100 μmol PDTC or 5 μmol MK-2206 were transfected with HPV E6 or NC and protein was extracted after 72 h. Western blot results suggested that pretreatment with specific inhibitors suppressed the activation of NF-κB and Akt signalings caused by E6 oncogene (Fig. 3h, i), implying that the effect of E6 oncogene on HNSCC cells was dependent on these signaling pathways.

### Relative activation of NF-κB and Akt pathways in HPV positive HNSCC samples

**Fig. 4:**
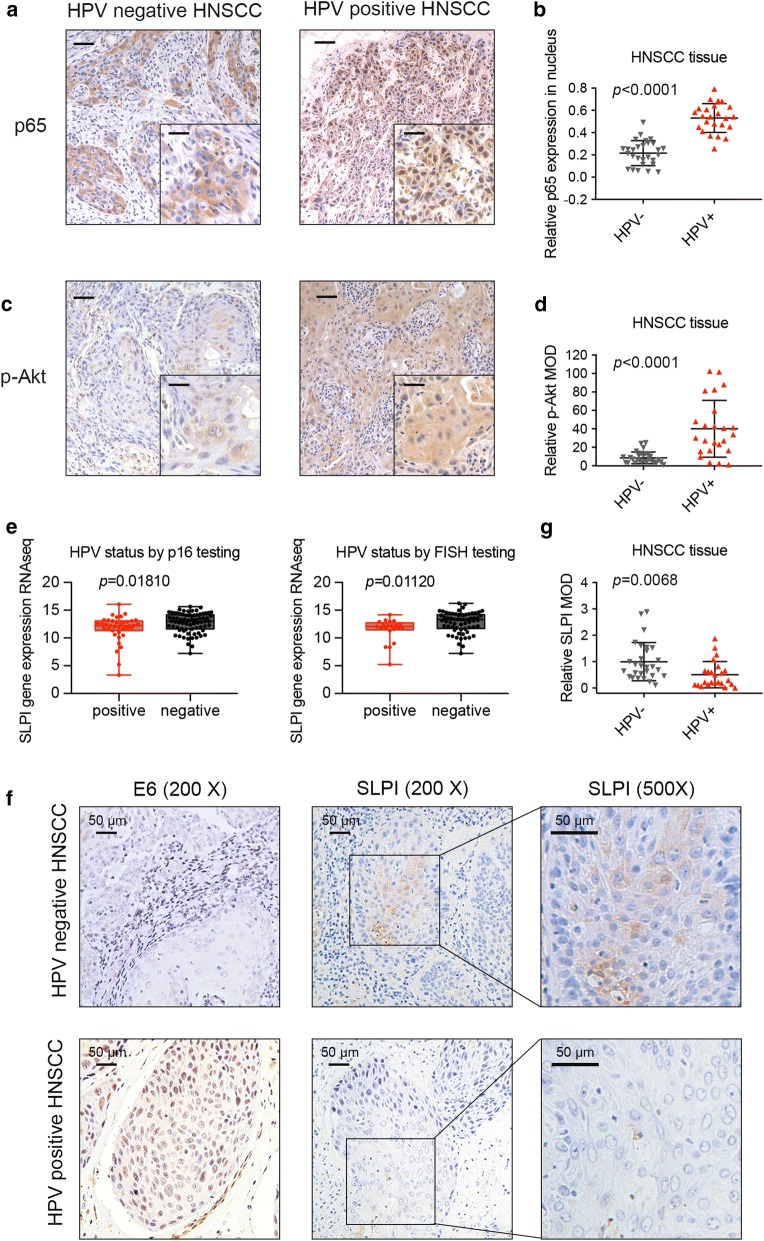
Relative activation of NF-κB and Akt pathways and SLPI downregulation in HPV positive HNSCC. **a**, **b** The proportion of p65 expressed in the cell nucleus in HPV positive tissues was significantly higher than that in HPV negative tissues. **c**, **d** The expression level of p-Akt in HPV positive HNSCC samples was much higher when compared to that in HNSCC samples without HPV infection (scale bar: main = 50 μm; insert = 15 μm). **e** mRNA expression level of SLPI was significantly decreased in HPV positive HNSCC samples (diagnosis both by FISH testing and p16 testing) according to the analysis results of mRNA data from the Cancer Genome Atlas (TCGA, http://xenabrowser.net). **f** Immunohistochemistry assay showed that HPV positive HNSCC tissues displayed a widespread expression of E6 oncogene, both in nuclear and cytoplasm while HPV negative tissues presented no E6 expression. Meanwhile, the staining intensity of SLPI was obviously lower in HPV positive HNSCC when compared to HPV negative HNSCC. **g** Statistical analysis of immunohistochemistry conducted on 24 HPV positive HNSCC tissues and 28 HPV negative tissues illustrated that SLPI protein level in HPV positive HNSCC was statistically lower than that in HPV negative ones

To further validate the activation of NF-κB and Akt pathways in HPV positive HNSCC, we implemented NF-κB and Akt-associated immunohistochemistry (IHC) staining on HNSCC samples. As suggested by previous studies [23–25], the relative expression and location of p65 represents the relative activities of NF-κB signaling. The intensity of p-Akt staining was utilized as an efficient marker for the relative activation of Akt pathway [26–28]. Therefore, we completed immunohistochemistry staining regarding p65 and p-Akt in HPV positive and negative HNSCC samples, evaluating the activation of NF-κB and Akt signalings. The intense nuclear staining of p65 and extensive expression of p-Akt in HPV positive HNSCC samples demonstrated the activation of NF-κB and Akt pathways (Fig. 4a, c). Moreover, statistical analysis (Fig. 4b, d) suggested the increased activities of NF-κB and Akt pathways in HPV positive HNSCC tissues when compared to HPV negative tissues.

### Downregulation of SLPI in HPV positive HNSCC

Before exploring the effect of SLPI on E6-expressing HNSCC cells, we first detected the relative level of SLPI in HNSCC samples with or without HPV infection. We found that the mRNA expression level of SLPI was further decreased in HNSCC samples with HPV infection (diagnosed both by FISH testing and p16 testing) compared to those without HPV infection according to the analytical results of mRNA data from the Cancer Genome Atlas (TCGA, http://xenabrowser.net) (Fig. 4e). To ensure the accuracy of this result, we conducted immunohistochemistry staining to detect the protein level of SLPI in 24 HPV positive HNSCC tissues and 28 HPV negative HNSCC tissues. As expected, positive E6 protein expression (both in the nucleus and cytoplasm) was observed in the HNSCC tissues infected with HPV while HPV negative tissues presented no E6 expression (Fig. 4f). Meanwhile, the protein expression of SLPI was obviously downregulated in HPV positive HNSCC tissues (Fig. 4f). Statistical analysis further suggested the relative lower SLPI protein level in HPV positive HNSCC (Fig. 4g). The above findings indicated that SLPI was further downregulated in HPV positive HNSCC tissues, implying that it may participate in the pathological process in HPV-mediated cancer progression.

### Exogenous SLPI can be internalized into HNSCC cells

**Fig. 5:**
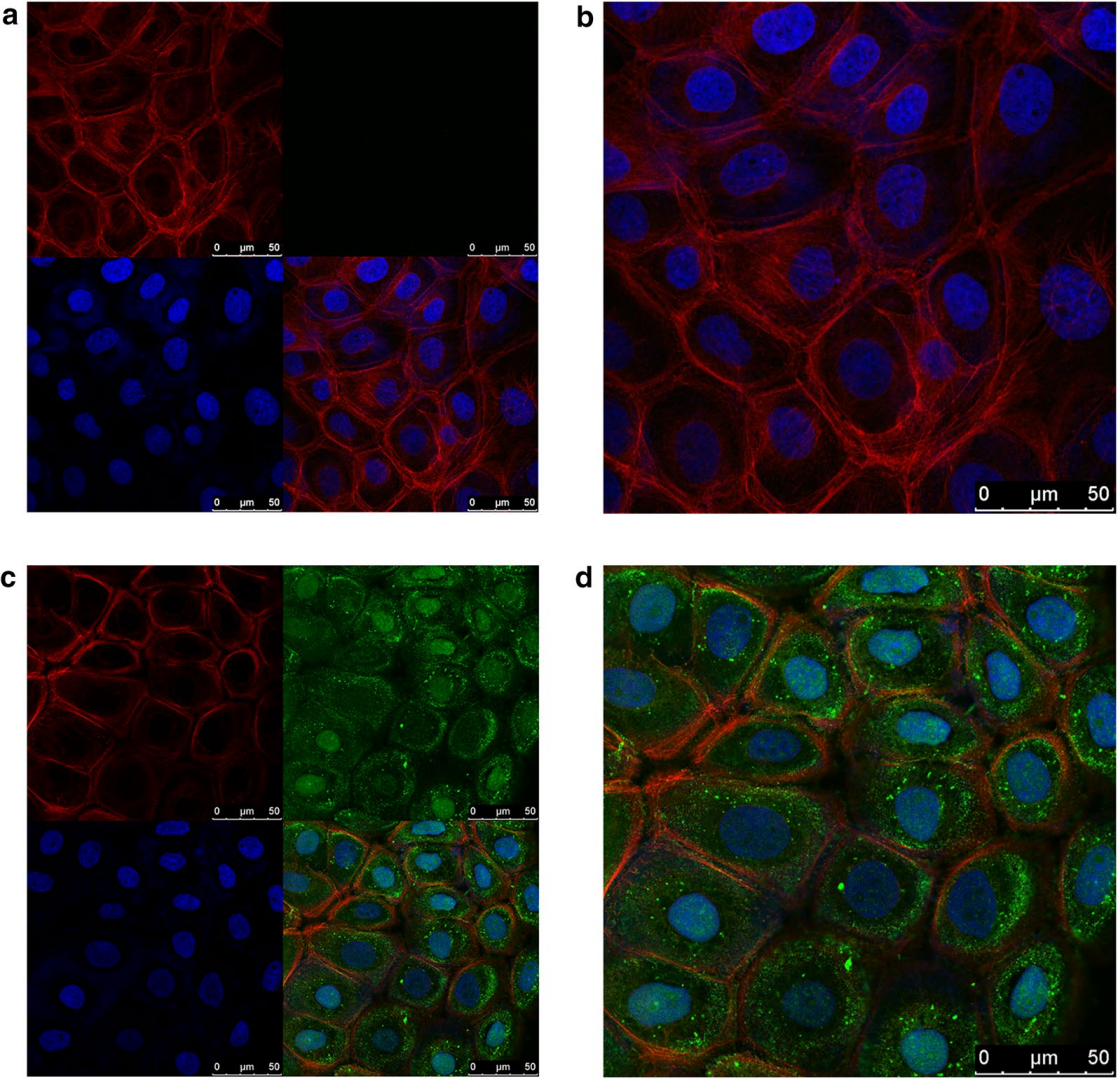
Exogenous SLPI could get internalized into HNSCC cells. **a**, **b** The images of HN4 cells incubated without exogenous SLPI protein. **c**, **d** The images of HN4 cells incubated with 40 μg/mL exogenous SLPI protein for 1 h. Cell nucleus was stained with DAPI (blue). Cytoskeleton was stained with phalloidine (red). SLPI was stained with FITC secondary antibody (green) (scale bar: 50 μm)

Since we utilized exogenous SLPI to investigate its potential effect on HNSCC cells, we first determined whether exogenous SLPI could get internalized into the cells. A TCS SP2 laser-scanning confocal microscope (Leica Microsystems, Germany) was used to observe the localization of SLPI in HN4 cells by immunofluorescent assay. As manifested in Fig. 5, exogenous SLPI was absorbed into cells and some entered the nuclei. Our results were in consistent with previous studies showing that SLPI could localize to the nuclei of activated human neutrophils [29]. Furthermore, another study found that SLPI could enter the monocytes, localizing to the cytoplasm and nucleus [30]. It was suggested that the ability of SLPI to enter the nucleus may be due to the cationic nature of this protein and its potential to interact with the predominantly negatively charged cell membrane. Additionally, its relatively small size may allow it to passively diffuse into the nucleus. It could be hypothesized that SLPI contains its own unique protein transduction domain. To elucidate more detailed underlying mechanisms, further investigations are still needed in the future.

### Exogenous SLPI remarkably inhibits HPV E6-mediated HNSCC cell proliferation and migration

**Fig. 6:**
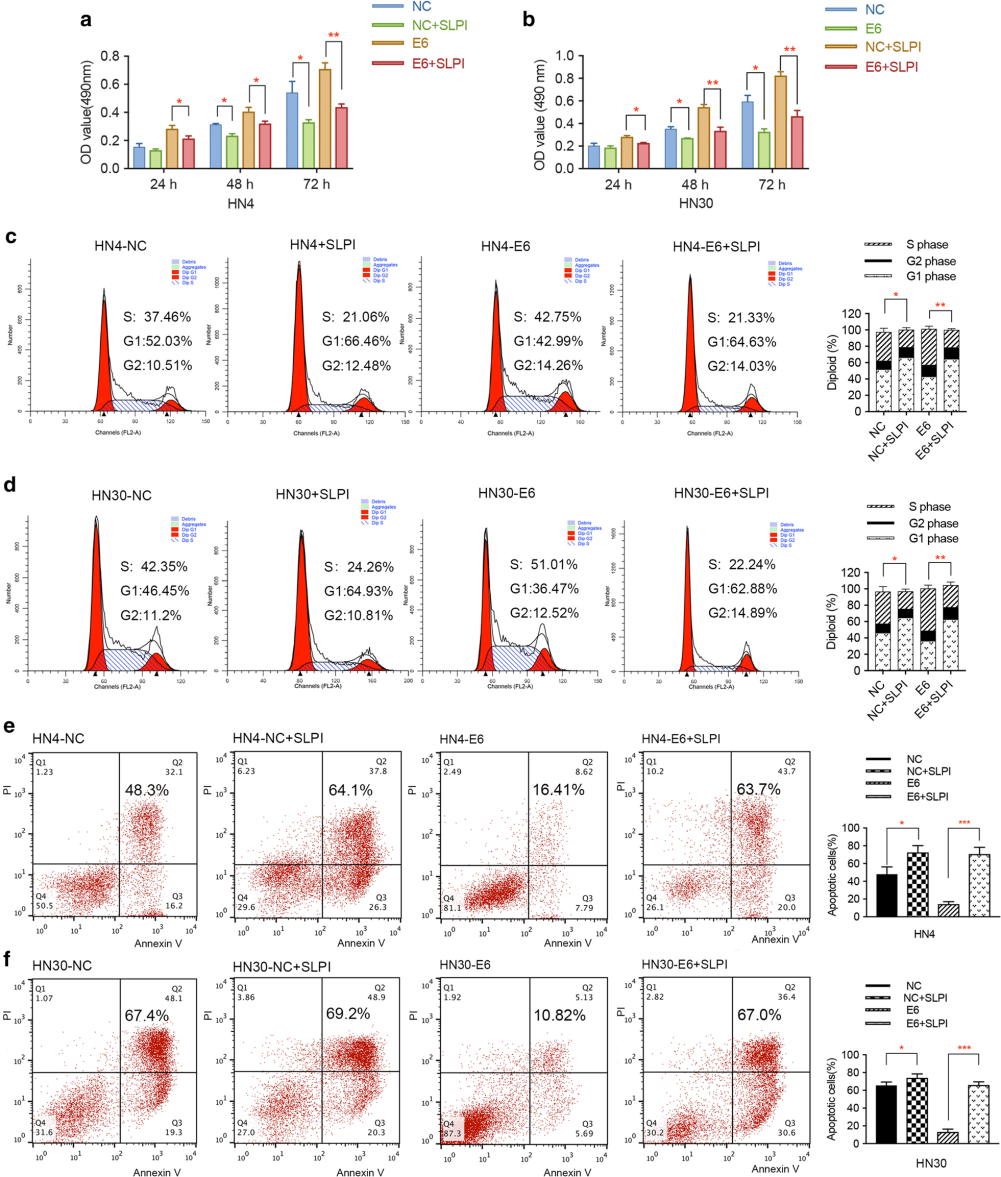
Exogenous SLPI inhibits cell growth and induces apoptosis activities in E6 positive HNSCC cells. **a**, **b** MTT assay revealed that exogenous SLPI significantly suppressed HPV E6 positive or E6 negative HNSCC cells proliferation. **c**, **d** Exogenous SLPI affected the cell distribution in HNSCC cells with or without E6 expression, mainly manifested by decreasing cells in S phase and increasing cells in G1 phase. **e**, **f** Apoptosis assay results suggested that apoptosis resistance of E6-expressing HNSCC cells was functionally reversed by SLPI treatment *P < 0.05. **P < 0.01. ***P < 0.001

**Fig. 7:**
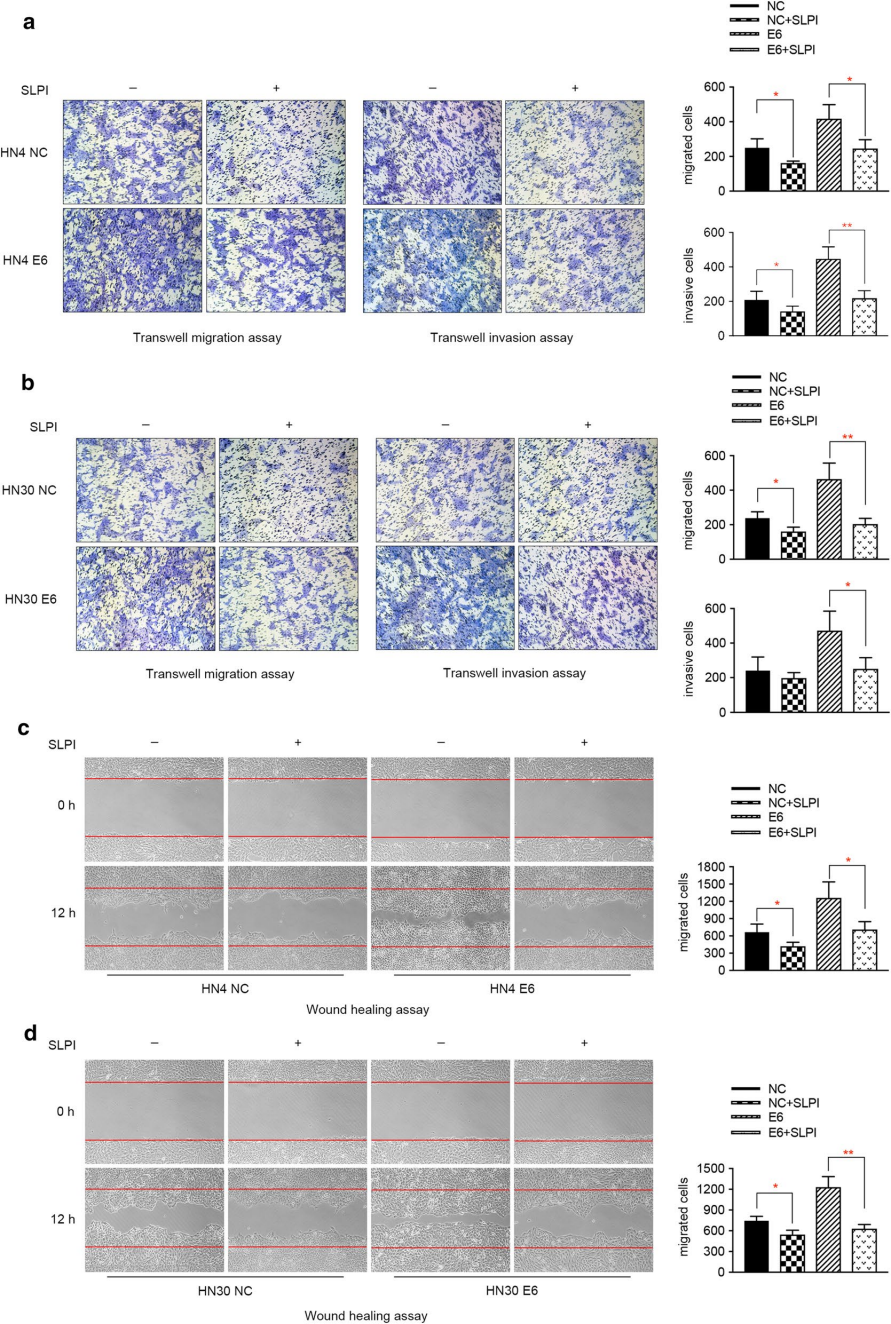
Exogenous SLPI remarkably inhibited the migration and invasion of HPV E6-expressing HNSCC cells. **a**, **b** Transwell migration and invasion assay demonstrated that SLPI could inhibit the migrating and invasive abilities of HPV E6 positive and E6 negative HNSCC cells. **c**, **d** Wound healing assay revealed the inhibitory role of SLPI in HNSCC cells migration. *P < 0.05. **P < 0.01

To investigate the biological effects of SLPI protein on HPV E6 positive or HPV negative cells, exogenous SLPI protein was added into the cell culture at a final concentration of 40 μg/mL and cell viability was analyzed at 24, 48 and 72 h by MTT assay. We found that SLPI notably inhibited the proliferation of E6 positive HNSCC cells compared to the control group (Fig. 6a, b). Further analysis revealed that SLPI could reduce the number of cells in S phase and induce cell apoptosis in both E6 positive and E6 negative HNSCC cells (Fig. 6c–f). Furthermore, as suggested in Fig. 7a–d, exogenous SLPI significantly suppressed the migrating and invasive abilities of tumor cells. More importantly, when comparing the inhibitory effect of SLPI on HNSCC cells with or without HPV E6 expression, we found that the suppressive effect on cell growth and metastasis was more evident in E6-expressing HNSCC cells, implying that specific mechanisms underlying SLPI-mediated E6 positive HNSCC progression may exist.

### Exogenous SLPI reverses E6-mediated activation of NF-κB and Akt signaling pathways in E6-expressing HNSCC cells

**Fig. 8:**
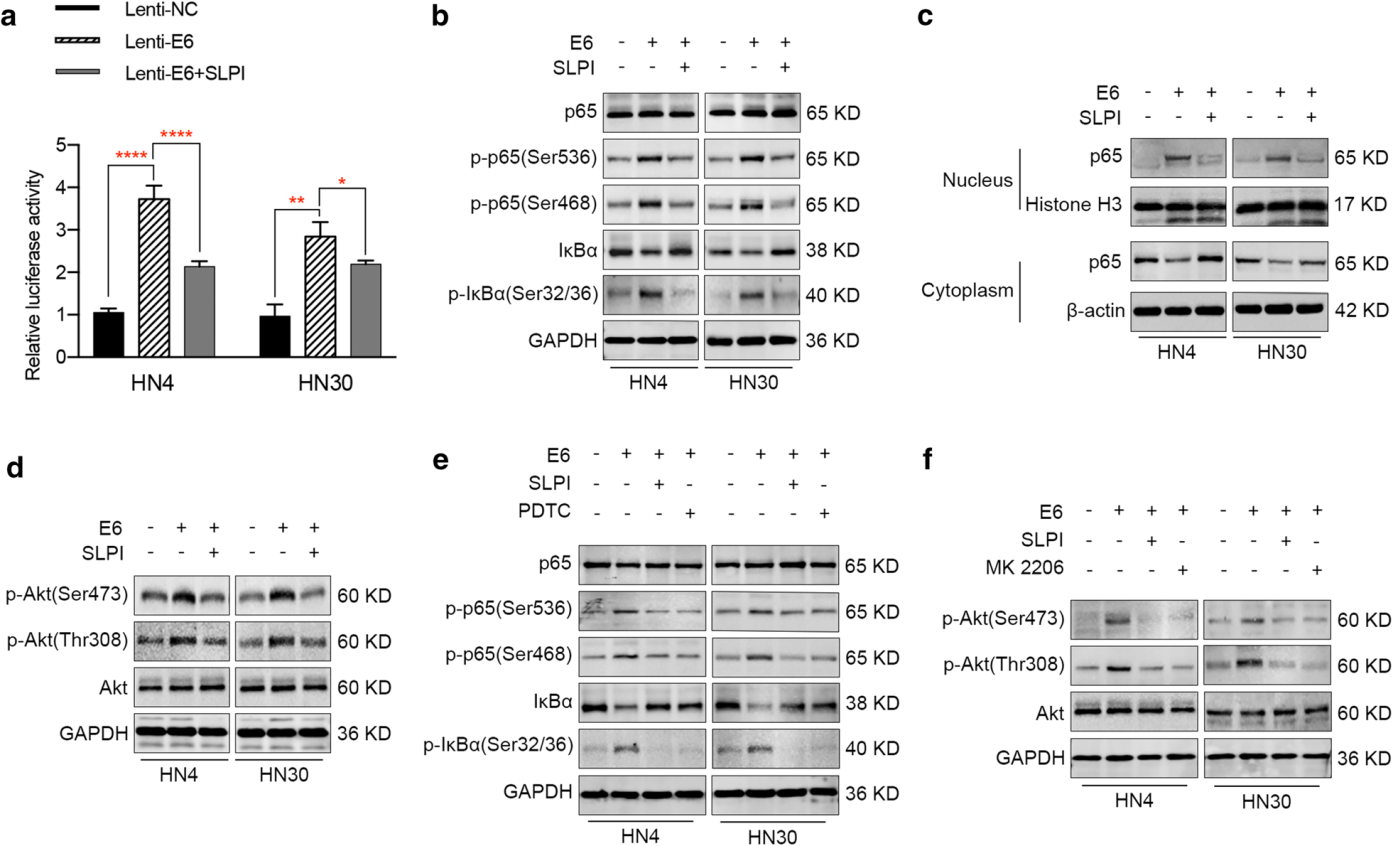
Exogenous SLPI reverses E6-mediated activation of NF-κB and Akt pathways in E6-expressing HNSCC cells. **a** NF-κB luciferase reporter assay demonstrated that administration of SLPI abolished the increase of NF-κB activities caused by E6 oncoprotein in HNSCC cells. **b** Western blot analysis showed that SLPI presented a suppressive effect on the phosphorylation of p65 and IκBα, and prevented the degradation of IκBα. **c** Nuclear and cytoplasmic proteins were extracted from cells and it was demonstrated that SLPI inhibited the translocation of p65 from the cytoplasm to the nucleus caused by E6 oncogene in HNSCC cells. **d** SLPI could reverse E6-mediated activation of Akt signaling pathway. **e**, **f** Specific inhibitors of NF-κB (PDTC, Beyotime) and Akt pathways (MK-2206, Selleck) were utilized to demonstrate that the effect of SLPI on HPV E6-expressing HNSCC cells may be obtained by mediating NF-κB and Akt pathways. *P < 0.05. **P < 0.01. ****P < 0.0001

**Fig. 9:**
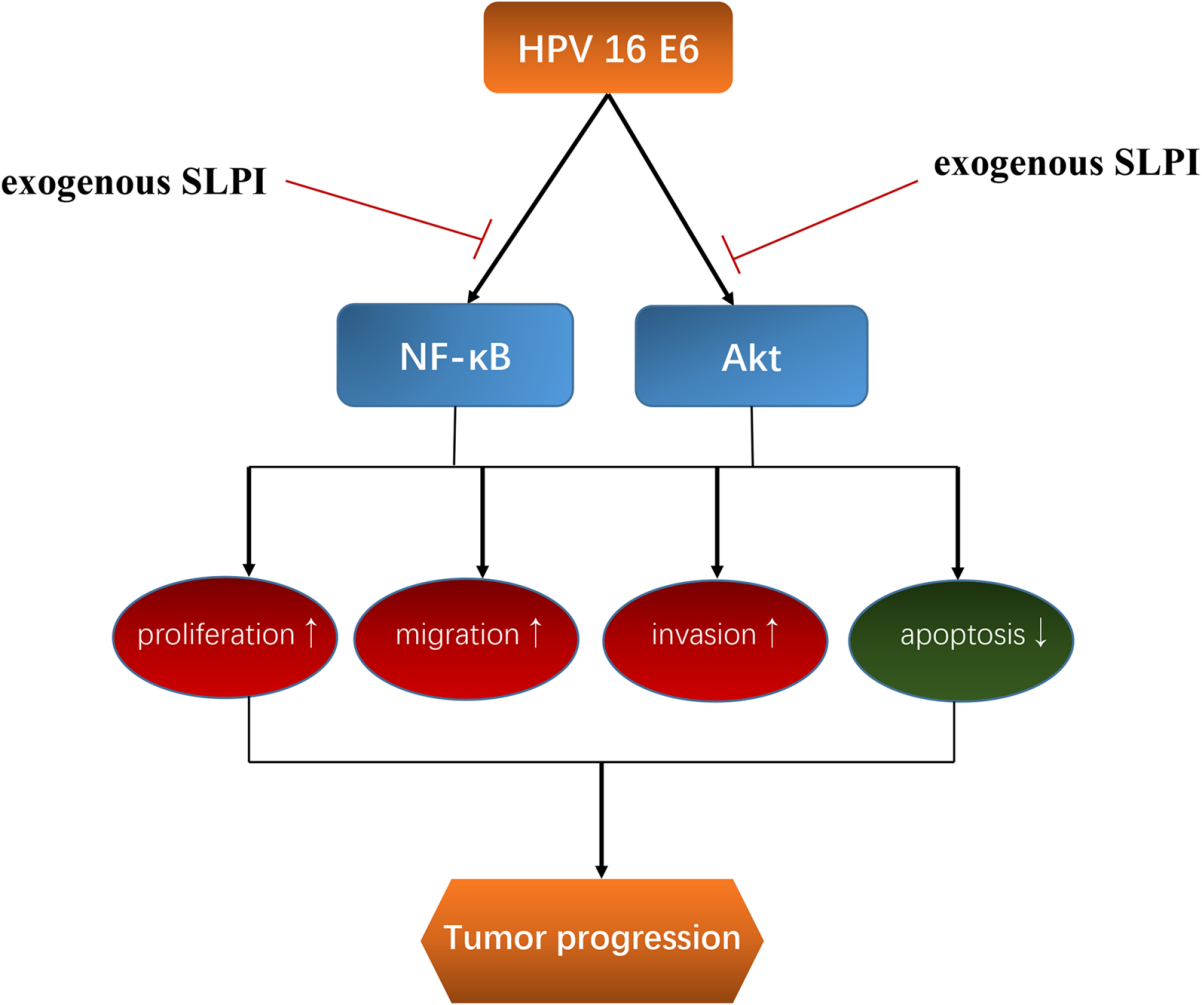
Schematic representation of the working model. The modal indicates that exogenous SLPI may affect HPV-E6 expressing HNSCC progression by inhibiting the activation of NF-κB and Akt pathways

In previous studies, SLPI was shown to negatively regulate the activation of NF-κB signaling pathway [31, 32]. Considering the participation of NF-κB signaling pathway in E6-mediated HNSCC progression, we investigated whether SLPI could reverse the E6-induced activation of NF-κB signaling pathway. The transcriptional activity of NF-κB was evaluated using a luciferase reporter gene assay. As shown in Fig. 8a, administration of SLPI abolished the increase of NF-κB activities caused by E6 oncoprotein, suggesting an inhibitory role of SLPI on NF-κB activation. Meanwhile, western blot analysis showed that SLPI had a suppressive effect on the phosphorylation of p65 and IκBα, and prevented the degradation of IκBα (Fig. 8b). Moreover, we also separated cytoplasmic and nuclear proteins from the E6-expressing HNSCC cells (HN4 and HN30) treated with or without SLPI, and western blot analysis revealed that SLPI inhibited the translocation of p65 from the cytoplasm to the nucleus caused by E6 protein (Fig. 8c). In addition, we also intended to identify whether SLPI could inhibit E6-induced activation of Akt signaling pathway. Fortunately, we found that SLPI treatment significantly decreased the phosphorylation of Akt in E6-expressing HN4 or HN30 cells (Fig. 8d). To further determine whether the effects of SLPI on HNSCC are really dependent on NF-κB and Akt signaling, we treated tumor cells with 40 μg/mL SLPI, 100 μmol PDTC or 5 μmol MK-2206 and corresponding protein changes were detected. As shown in Fig. 8e, f, the relative activities of NF-κB and Akt pathways were similar in cells treated with SLPI or specific inhibitors, implying that the inhibitory effect of SLPI on E6-expressing HNSCC cells was dependent on mediating these two pathways. Overall, it came to the conclusion that SLPI could interfere with multiple pathways through which E6 oncogene performed its tumor promoter role in HNSCC, suggesting the potential therapeutic effect of SLPI on E6 positive HNSCC. The overall schematic representation of the working model was illustrated in Fig. 9.

## Discussion

HPV has increasingly been recognized as an essential risk factor for HNSCC initiation and progression and the two HPV-specific oncogenes E6 and E7 could always lead to the malignant transformation of cancer cells. However, the detailed biological roles of E6 oncogene have not been well elucidated in HNSCC. In this study, we constructed HPV E6-expressing HN4 and HN30 cells and explored the impact of E6 oncogene on HNSCC development. Consistent with its tumor promoter role, we found that E6 significantly facilitated HNSCC progression through promoting the proliferation, cell cycle progression, apoptosis resistance, migration and invasion of HNSCC cells.

Apart from highlighting the malignant transformation of HNSCC phenotypes by E6 oncogene, we further intended to investigate the underlying mechanisms. NF-κB is a proinflammatory transcription factor that plays a vital role in the initiation and progression of multiple cancers, including HNSCC [33]. Normally, NF-κB remains in an inactive form in the cytoplasm and once activated by a variety of stimulants, such as cytokines, lipopolysaccharide, bacterial or viral infection, NF-κB could translocate to the nucleus and then modulate the expression of downstream target genes that participate in a number of cellular functions, including cell proliferation, apoptosis, cell migration and angiogenesis [34]. HPV infection has been reported to be closely associated with NF-κB and this process may be regulated by HPV 16 oncogenes. However, conflicting evidence exists regarding whether E6 oncogene stimulates [35–37] or suppresses [38] NF-κB activation. From our perspective, the contradictory effect of E6 on the same signaling pathway may be explained by the theory that NF-κB pathway activation may be dependent on the cell type and signal context [39]. The present study clarified how HPV E6 regulated NF-κB in HNSCC cells and suggested that E6 oncogene may act as a tumor promoter by activating NF-κB signaling pathway. However, the detailed mechanism regarding how E6 mediates the activity of NF-κB signaling pathway has not been fully explored. One possible explanation is that NF-κB and p53 were found to directly compete for binding to the transcriptional coactivator CBP/p300 and the degradation of p53 by E6 may result in the activation of NF-κB in E6-expressing cells [40]. Other studies also revealed that p53 was able to suppress the transcriptional activity of NF-κB and may account for the indirect effect of E6 oncoprotein on NF-κB pathway [41]. Hence, further studies should be implemented to fully elucidate the mechanism by which E6 regulates the activity of NF-κB signaling pathway in HNSCC in the future.

Based on previous studies concerning the involvement of HPV in Akt [42–44] signaling pathway, we investigated whether Akt signaling was also involved in E6 oncogene-mediated HNSCC progression. As a result, we found that abnormal expression of E6 protein in HNSCC cells promoted the phosphorylation of Akt, which indicated the activation of Akt pathway. For the underlying mechanism of HPV16 E6-induced Akt activation, Spangle et al. found that E6 protein could consistently activate the receptor protein tyrosine kinases including epidermal growth factor receptor, which is one of the upstream proteins of the PI3K/Akt pathway [42]. Another study stated that the inactivation of PTEN by E6 led to the increased p-Akt and enhanced cell proliferation [45]. Therefore, we concluded that HPV E6 oncogene participated in multiple essential pathways in HNSCC progression. More importantly, it is well known that some signaling pathways such as PI3K/Akt pathway, could cause drug resistance in cancer and a recent study illustrated that the activation of PI3K/Akt pathway by E6 oncogene led to the resistance to cisplatin in HPV-associated lung cancer [22]. Therefore, modulation of these signaling pathways has therapeutic significance in HPV E6 positive HNSCC [42].

SLPI has previously been reported by a good number of studies to influence the progression of various cancers, either as a tumor promoter or tumor suppressor. For example, SLPI promoted proliferation and survival of ovarian cancer cells through partnering with prgn [46]. Also, overexpression of SLPI was closely associated with invasion and metastasis of gastric cancer by regulating p53, bcl-2 and caspase-8 expression [11]. In addition, SLPI was shown to stimulate ovarian cancer invasion and this function was partly mediated by its serine protease inhibitory activity attenuating MMP-9 release [47]. In addition to the tumor-promoting roles of SLPI, it has also been demonstrated to inhibit tumor progression in a variety of cancer types. In breast cancer cells, SLPI exerted pro-apoptotic and cell cycle-arrest effects [9]. Furthermore, an in vivo experiment revealed that inoculation of SLPI overexpressing mammary tumor cells decreased tumor growth in mice, implying a potential SLPI-dependent immunotherapy method [48]. More importantly, our preliminary study demonstrated that SLPI acts as a tumor suppressor and functionally influences the proliferation and apoptosis of HNSCC cells [13]. From our perspective, the contradictory role of SLPI in tumor progression might be due to the different tumor origins and pathological types and the fact that SLPI regulates distinct targets in different cancers may also account for the opposing effect of SLPI on tumor progression. Due to the context-dependent function of SLPI, we wondered whether SLPI still maintains its inhibitory role in HNSCC cells that have been transformed by E6 oncogene. By treating E6-expressing cells with exogenous SLPI, we found that SLPI remarkably reversed the E6-induced phenotypes in HNSCC. For the underlying mechanisms involved in the suppression of tumor progression by SLPI, numerous studies have focused on the NF-κB signaling pathway. SLPI has been reported to inhibit LPS-induced NF-κB activation by displacing or inhibiting NF-κB binding to its promoter regions in target genes [30]. Furthermore, another study found that when SLPI entered cells, it could rapidly localize to the cytoplasm and nucleus, competing with p65 for binding to the NF-κB binding cites so as to affect NF-κB activation [49]. In addition, SLPI prevented the degradation of IκBα, thus decreasing the translocation of p65 from cytoplasm to nucleus and resulting in the inhibition of NF-κB activation [50]. Based on these reports, we hypothesized that the inhibitory effect of SLPI on E6 positive HNSCC progression was partly achieved through preventing the activation of NF-κB caused by E6 oncogene. In this study, NF-κB luciferase reporter assay and western blot analysis consistently demonstrated that SLPI suppressed the NF-κB signaling pathway activation caused by E6 oncogene. Since HPV E6 mediated multiple pathways in cancer progression, we then attempted to explore whether SLPI was also involved in Akt signaling though no relevant studies exist. Fortunately, the results showed that SLPI could reverse E6-induced activation of Akt pathway in HNSCC. The above data indicated that SLPI could inhibit proliferation, induce cell cycle arrest, and promote apoptosis activities in E6 positive HNSCC cells and abolish multiple cancer-related pathways activation caused by E6 oncogene. Since it has been suggested that the inhibition of multiple signalings is necessary for HPV-associated cancers, we supposed that SLPI may serve as a candidate therapy agent [42]. Meanwhile, the fact that SLPI presented at a significantly lower level in HPV positive HNSCC tissues compared to those tissues without HPV infection laid a foundation for its therapeutic potential. However, more comprehensive experiments should be implemented in the future.

## Conclusions

In summary, this study showed that HPV 16 E6 oncogene was expressed in HPV positive HNSCC tissues and highlighted the functional role of E6 oncogene in the growth, migration and invasion of HNSCC cells. Moreover, we validated that E6 protein regulated the biological behaviors of HNSCC cells by collectively activating NF-κB and Akt pathways collectively. Then, exogenous SLPI could functionally inhibit the E6-induced HNSCC progression through reversing the activation of NF-κB and Akt signalings mediated by E6 oncogene. Since E6 oncogene accounts for the carcinogenesis of HPV-induced HNSCC to a great extent, we proposed that therapy based on reversing E6-mediated biological processes may be worthwhile to explore.

## Data Availability

The datasets used in this study are available from the corresponding author upon reasonable request.

## Abbreviations

HPV: human papillomavirus
HNSCC: head and neck squamous cell carcinoma
SLPI: secretory leukocyte protease inhibitor
DMSO: dimethyl sulfoxide
rhSLPI: recombinant human SLPI
